# Is the acquired hypothyroidism a risk factor for developing psychiatric disorders?

**DOI:** 10.3389/fpsyt.2024.1429255

**Published:** 2024-07-19

**Authors:** Norma Osnaya-Brizuela, Armando Valenzuela-Peraza, Daniel Santamaría-del Ángel, Yuliana García-Martínez, Jorge Pacheco-Rosado, Gilberto Pérez-Sánchez, Karla Sánchez-Huerta

**Affiliations:** ^1^ Laboratorio de Neurociencias, Subdirección de Medicina Experimental, Instituto Nacional de Pediatría, Ciudad de México, Mexico; ^2^ Departamento de Fisiología “Mauricio Russek”, Escuela Nacional de Ciencias Biológicas, Instituto Politécnico Nacional, Ciudad de México, Mexico; ^3^ Laboratorio de Psicoinmunología, Instituto Nacional de Psiquiatría Ramón de la Fuente Muñíz, Ciudad de México, Mexico

**Keywords:** acquired hypothyroidism, depression, anxiety, TSH, triiodothyronine (T3), thyroxine (T4), levothyroxine, L-T4

## Abstract

Hypothyroidism is a prevalent thyroid condition in which the thyroid gland fails to secrete an adequate amount of thyroid hormone into the bloodstream. This condition may develop due to genetic or acquired factors. The most frequent cause of acquired hypothyroidism is chronic autoimmune thyroiditis, also known as Hashimoto’s disease. Acquired hypothyroidism is diagnosed when patients present with overt hypothyroidism (also known as clinical hypothyroidism), as they exhibit increased TSH and decreased T_3_ and T_4_ serum levels. This article examines the prevalence of psychiatric disorders among patients diagnosed with acquired hypothyroidism with or without Levothyroxine treatment. We discuss the available evidence indicating that acquired hypothyroidism may be a risk factor for psychiatric disorders, and the effectiveness of thyroid treatment in relieving psychiatric symptoms. Additionally, we provide critical details on thyroid hormone cutoff values reported in the literature, their potential clinical importance, and their correlation with psychiatric symptoms. Finally, we examined the various mechanisms by which acquired hypothyroidism can lead to depression. The high rate of comorbidity between hypothyroidism and psychiatric disorders deserves special attention, indicating the importance of consistent monitoring and timely identification of psychiatric symptoms to prevent disease exacerbation and facilitate therapeutic management. On the other hand, several mechanisms underlie the strong association between depression and acquired hypothyroidism. Deeper research into these mechanisms will allow knowledge of the pathophysiology of depression in patients with acquired hypothyroidism and will provide clues to design more precise therapeutic strategies for these patients.

## Highlights

- Acquired hypothyroidism increases the risk of depression and anxiety in adult patients with or without L-T4 treatment.
- Acquired hypothyroidism may lead to depression by multiple mechanisms, including alterations in serotonergic neurotransmission or the HPA axis, changes in adult hippocampal neurogenesis or brain region integrity, activation of systemic pro-inflammatory processes or neuroinflammation, or triggering of the kynurenine pathway.
- Several lines of research should be undertaken to investigate whether acquired hypothyroidism is a risk factor for the development of schizophrenia or bipolar disorder, or for the development of psychiatric disorders in children and adolescents.

## 1 Introduction

Hypothyroidism is a medical condition resulting from inadequate production of thyroid hormones (THs), often caused by thyroid gland dysfunction. According to Usman et al. (2023), hypothyroidism is diagnosed when thyroid stimulating hormone (TSH), triiodothyronine (T_3_), and thyroxine (T_4_) are >4.94 μIU/mL, <1.71 pg/mL, and <0.7 ng/dL, respectively (1). This condition results in clinical complications and disturbances in the physiological maintenance of bodily functions (2). Hypothyroidism can manifest because of either genetic or acquired factors. The latter can affect individuals during childhood, adolescence, or adulthood, and develops from sources other than genetic ones (2, 3). The most frequent cause of acquired hypothyroidism is chronic autoimmune thyroiditis, more commonly known as Hashimoto’s disease. In addition to other causes, such as iodine deficiency, thyroidectomy, radioactive iodine therapy, radiation (4), viral infections (5), and drug use (6). In low-income countries, iodine deficiency is the leading cause of acquired hypothyroidism, whereas, in more developed countries (where foods are enriched and fortified with iodine), autoimmune thyroiditis is the predominant cause (6).

Acquired hypothyroidism is diagnosed when patients present with overt hypothyroidism (also known as clinical hypothyroidism), as they exhibit increased TSH and decreased T_3_ and T_4_ serum levels (TSH >4.94 μIU/mL; T_3_ <1.71 pg/mL; T_4_ <0.7 ng/dL). The prevalence of this disease may differ based on gender, age, or geographic location (7, 8). In the United States, in adolescent and adult populations, the prevalence of acquired hypothyroidism ranged between 2.1 to 6.1% depending on the criteria for defining hypothyroidism. Moreover, a higher occurrence in women was reported (8). In European countries, this prevalence was 0.37% with predominance in females (9). In Mexico, it was reported between 1.2–1.8% in the adult population (10, 11).

The close relationship between hypothyroidism and psychiatric symptoms has been the subject of extensive research. The evidence indicates an association between thyroid function and psychiatric disorders, including anxiety, depression, bipolar disorder, and schizophrenia (12, 13). Most clinical studies and previous reviews have focused on describing thyroid alterations in patients with psychiatric disorders, and the effects of psychiatric drugs on the thyroid endocrine system (12, 14, 15). In contrast, there are fewer scientific studies regarding the prevalence of psychiatric disorders in patients initially diagnosed with acquired hypothyroidism. Additionally, only a minority of investigations examine the effectiveness of Levothyroxine (L-T_4_) treatment in mitigating or alleviating psychiatric disorders in these kinds of patients.

A pioneering study, published in the 60´s decade, showed that hypothyroidism could be causing psychiatric disorders. The study reported eight patients with both concurrent hypothyroidism and mental illness; six of them recovered with treatment consisting of consumption of thyroid gland or dried thyroid tissue and showed normal mental status in the medium term (from 2 to 12 years after the event) (16). It was suggested that hypothyroidism acts as an inducer of mental changes and that thyroid medication could be relevant to the recovery of mental health. Recent evidence continues supporting this notion, the studies were compiled in this review to answer whether the acquired hypothyroidism acts as a risk factor for psychiatric disorders in patients with or without thyroid medication, and whether the L-T_4_ treatment is successful in alleviating the psychiatric illness. In addition, we briefly address the possible mechanisms underlying the establishment of depression in patients with acquired hypothyroidism providing key points that should be considered for future research.

## 2 Methods

The search criteria, including the literature inclusion and exclusion criteria, as well as the limitations, are described in detail in the **Supplementary Material**.

## 3 Depression in patients with acquired hypothyroidism

Depression is classified as a mood disorder that presents with persistent sadness, emptiness or irritable mood, loss of interest in daily activities, hopelessness, difficulty experiencing pleasure, as well as disturbances in sleeping and eating (17, 18). According to the World Health Organization (WHO), one in eight people worldwide has a mental disorder, and depression affects 280 million people (19).

Depressive symptoms have a high prevalence in patients with hypothyroidism, and the study of this close relationship between the two conditions has aroused great interest in both the psychiatric and endocrinological fields. One of the greatest concerns in patients with acquired hypothyroidism is the imminent risk of developing depression, which will worsen the patient’s condition and its therapeutic management. Based on different studies (8–11), the prevalence of acquired hypothyroidism is reported between 0.3 and 6.1%, whereas depression can afflict up to 18% of the population, according to Lee’s findings (2023) (20). Even more interestingly, patients with acquired hypothyroidism have a high prevalence of depression reaching 79.2% in patients with hypothyroidism without treatment (21), between 12.1 and 36.7% in patients under L-T_4_ treatment (1, 22, 23), and between 33.3 and 66.7% in patients who had developed an euthyroid state after L-T_4_ treatment (See **Table 1**) (24, 25). This high prevalence of depression in patients with acquired hypothyroidism, with or without thyroid treatment, is in line with a higher risk of developing this psychiatric illness.

**Table 1:** Prevalence of depression in patients with acquired hypothyroidism without or with L-T_4_ treatment.

Prevalence of depression in patients with acquired hypothyroidism	Population’s characteristics	Psychiatric test	Reference
79.2%	Female patients with acquired hypothyroidism without thyroid medication.	Primary care evaluation of mental disorders (PRIME MD)	Guimaraes et al., 2009 (21)
12.1% (*)	Patients with diagnosis of acquired hypothyroidism under L-T_4_ treatment.	Diagnostic and statistical manual of mental disorders (DSM-III-R)	Bunevicius et al., 1999 (23)
33.9%	Patients with diagnosis of acquired hypothyroidism under L-T_4_ treatment.	Patient health questionnarie-9 (PHQ-9)	Mohammad et al., 2019 (22)
36.7%	Patients with diagnosis of acquired hypothyroidism under L-T_4_ treatment.	PHQ-9	Usman et al., 2023 (1)
66.7% (*)	Patients with diagnosis of acquired hypothyroidism who had developed euthyroid state after treatment with L-T_4_.	Beck depression inventory (BDI)	Talaei et al., 2017 (24)
54.5% (patients with 1 year of follow-up) 41.7% (patients with 5 year of follow-up) 33.3% (patients with 10 years of follow-up)	Patients with diagnosis of acquired hypothyroidism who had developed euthyroid state after treatment with L-T_4_.	BDI	Gunes, 2020 (25)

*These values were calculated employing the article’s data. In Bunevicius et al. (1999), the ratio was 4/33. In Talei et al. (2017), the ratio was 116/174.

### 3.1 Depression in patients with acquired hypothyroidism without thyroid medication

Ittermann et al. (2015) showed that patients with acquired hypothyroidism, not taking medication, have 2.10 (95% CI, 0.86–5.11) or 2.32 (95% CI, 1.28–4.21) times higher risk of developing major depressive disorder according to data obtained by the Munich composite international diagnostic interview (M-CIDI) or Beck depression inventory II (BDI-II), respectively (26). Interestingly, this risk seems to be more if only women are evaluated. Guimaraes’ research in women with overt hypothyroidism (TSH>4 μIU/mL and free T_4_<0.7 ng/dL) found that they face 8.7 times (95% CI, 2.56–29.50) higher risk of experiencing depression than euthyroid women (21). The authors used Spitzer et al.’s (1994) PRIME-MD test (27) instead of the Beck depression inventory (BDI) for the assessment of depressive symptoms (21). Regarding the severity of depression, the literature shows that depressive symptoms were more severe in patients with overt hypothyroidism than control subjects or patients evaluated after receiving L-T_4_ treatment, based on elevated scores in the Hamilton depression rating scale (HDRS) and BDI test (28, 29).

### 3.2 Depression in patients with acquired hypothyroidism with thyroid medication

Regarding patients with acquired hypothyroidism under thyroid medication, Thvilum et al. (2014) found that, after the diagnosis of hypothyroidism, these patients were more likely to develop depression and require antidepressant treatment (HR: 1.30, 95% CI: 1.15–1.47) (30). The severity of their depressive stage was predominantly mild or moderate-severe. Mohammad et al. (2019) showed that depression was moderate in 10.7%, moderate-severe in 19.6%, and severe in 3.6% of these patients (22). In line, Usman et al. (2023) showed that such patients experienced mild, moderate, moderate-severe, and severe depressive symptoms in 44.4%, 33.3%, 16.7%, and 5.6% of cases, respectively (1). Both authors determined the severity of depression using the patient health questionnaire-9 (PHQ-9) (1, 22).

Clinical studies in patients receiving L-T_4_ treatment have been also useful to study the relationship between TSH levels and depression severity, suggesting a positive correlation between elevated TSH levels and more severe depression (21, 24). Talaei et al. (2017) proposed a cut-off value of TSH for the evaluation and consideration of antidepressant treatment in patients with hypothyroidism treated with L-T_4_ (24). They showed that a TSH cut-off point of ≥2.5 mIU/L is associated with a depression diagnosis, displaying 89.6% sensitivity (95% CI, 82.6–94.5) and 87.9% specificity (95% CI, 76.7–95.0). Additionally, the study found that a cut-off point of ≥4mIU/L indicated severe depression with 80.5% (95% CI, 64.0–91.8) and 95% (95.6% CI, 90.8–98.4) sensitivity and specificity, respectively. It’s worth noting that the patients with hypothyroidism in Talaei et al.’s (2017) study were under treatment with L-T_4_, with a mean age of 45.5 ± 11.7 years, and had mean T_3_ and T_4_ values of 1.2 ng/mL and 8.4 pg/dL, respectively. In addition, BDI score above 10 was used as a criterion for depression diagnosis (24). A study conducted by Guimaraes et al. (2009) revealed that 65% of women (mean age of 53.6 years) with TSH levels ≥10 μIU/mL exhibited depressive symptoms and had a 3-fold (95% CI, 1.21–7.79) higher likelihood of developing depression compared to those with normal TSH levels (>0.3 and ≤4 μIU/mL) (21).

Finally, it can be inferred that there is a close association between thyroid dysfunction and the onset of depressive symptoms. Some studies have even delved into the connection between the hypothyroid condition and a higher incidence of suicidal behavior. In a meta-analysis conducted by Toloza et al. (2021), it was reported that patients with suicidal behavior have significantly lower levels of free T_3_ and total T_4_ as compared to patients without suicidal behavior (31). The authors clarify that research on the correlation between thyroid disorders and suicidal behavior is limited, warranting further investigation for improved understanding.

Thus, individuals with acquired hypothyroidism, whether treated or not, are more susceptible to depression, according to current evidence. It is important to consistently screen these patients for depressive symptoms to manage this comorbid psychiatric condition and prevent further complications.

## 4 Anxiety disorder in patients with acquired hypothyroidism

Anxiety disorder is characterized by excessive fear, anxiety, and related behavioral disturbances. Fear is the emotional response to a real or perceived imminent threat, whereas anxiety is the anticipation of future threats. Such anxious responses are disproportionate to the context or triggering stimuli and can significantly hinder a person’s daily life. Common symptoms of anxiety may comprise persistent worry, irritability, restlessness, difficulty concentrating, muscle tension, sleep disturbances, panic attacks, and avoidance of anxiety-provoking situations (18). Anxiety is a prevalent psychiatric disorder that affects 18.1% of people in the United States (32) and 301 million people worldwide (19). Interestingly, the presence of anxiety disorder has been reported in patients with acquired hypothyroidism, as evidenced by various studies in the adult population.

### 4.1 Anxiety in patients with acquired hypothyroidism without thyroid medication

Ittermann et al. (2015) found that individuals with a diagnosis of acquired hypothyroidism, with a mean age of 55 years and without L-T_4_ treatment, have a 3.98 (95% CI, 1.48–10.72) higher risk of experiencing anxiety (test: M-CIDI) (26). In addition, these patients had significantly higher scores for anxiety, as well as decreased attention and executive task performance, in comparison with control subjects (test: state-trait anxiety inventory (STAI)) (29, 33). These elevated scores for anxiety were also reported when patients with overt hypothyroidism were compared with patients under L-T_4_ treatment or with TSH values below 0.1 µIU/mL (test: Thyroid-related quality-of-life patient-reported outcome (ThyPRO)) (34).

### 4.2 Anxiety in patients with acquired hypothyroidism with thyroid medication

Thvilum et al. (2014) found that hypothyroid patients, even under L-T_4_ treatment, had an increased risk of developing anxiety and requiring anxiolytic treatment after diagnosis of hypothyroidism (HR: 1.27; 95% CI, 1.10–1.47) (30). Interestingly, it has been reported that even after becoming euthyroid with thyroid medication, patients had higher anxiety scores than controls at 1, 5, and 10 years of follow-up (test: Beck anxiety inventory (BAI)) (25). The patients experienced several symptoms, including hot flushes, weakness, tremors in the legs and hands, dizziness or drowsiness, palpitations, a feeling of loss of balance, becoming terrified, and flushing of the face (25). Additionally, it has been demonstrated that discontinuing thyroid medication can result in increased anxiety levels (29, 33).

The relationship between serum TSH levels and anxiety disorders has not been extensively studied. One report found that serum TSH levels and gender were the most significant predictive factors when anxiety was considered a dependent variable (34).

Overall, the literature suggests an association between hypothyroidism and anxiety, as supported by the higher risk of developing this disorder in patients with acquired hypothyroidism without or under L-T_4_ treatment. Discontinuation of thyroid medication in hypothyroid patients appears to be a risk factor for the development of anxiety in patients with this condition, making appropriate and timely therapeutic management and psychiatric care highly relevant.

## 5 Negative evidence about the relationship between acquired hypothyroidism and depression or anxiety

In contrast to other authors, Grabe et al. (2005) showed that patients with overt hypothyroidism did not differ from euthyroid controls in scores for anxiety and depression employing Zerssen complaint scale (2005) (35). The authors explain that hypothyroid patients may have a differential susceptibility to low thyroid hormone levels, with not all of them developing a psychiatric disorder.

In line, Gulseren et al. (2006) found that patients with overt hypothyroidism had similar scores for anxiety that control subjects, employing the Hamilton anxiety rating scale (HARS) (28). The authors explain that the causal relationship between thyroid dysfunction and anxiety is still speculative and that their findings need to be interpreted with caution due to the small sample size and that studies in larger populations are needed.

## 6 Schizophrenia or affective bipolar disorder in patients with acquired hypothyroidism

Schizophrenia is a mental disorder that is primarily characterized by a progressive decline in cognitive function and a range of psychiatric symptoms, such as hallucinations, delusions, and disorganized thinking (speech) (18, 36). On the other hand, bipolar disorder is a chronic mood disorder mainly characterized by a mixture of manic (bipolar mania), hypomanic, and depressive (bipolar depression) episodes (37, 38). Schizophrenia and bipolar disorder affect 24 and 40 million people worldwide, respectively (19), and both have been associated with hypothyroidism since the 1960s (39, 40).

In 2018, Sharif and colleagues found that individuals with hypothyroidism exhibited a significantly higher prevalence of schizophrenia (2.01%) compared to controls (1.25%). Nevertheless, the study was unable to establish a causal relationship between hypothyroidism and schizophrenia. One limitation of this study was that the etiology and type of hypothyroidism were not determined (41). In a separate study, Benros and colleagues (2011) found that a previous autoimmune disease increased the risk of schizophrenia by 29%, However, only a small number of patients presented with autoimmune thyroiditis (n=3), suggesting that there was no evidence of an association between autoimmune thyroiditis and the development of schizophrenia (42).

On the other hand, Thomsen et al. (2005) analyzed data from a Danish patient registry to investigate whether hospitalization for hypothyroidism increases the risk of developing bipolar disorder. They found that only 0.17% of hypothyroid patients were later hospitalized for bipolar disorder, which is comparable to the hospitalization rate for patients with nontoxic goiter or osteoarthritis (43).

Thus, there is insufficient evidence to conclude whether hypothyroidism is a risk factor for the development of schizophrenia or bipolar disorder. Therefore, longitudinal studies are needed to determine the incidence of these two disorders in patients initially diagnosed with acquired hypothyroidism.

## 7 Effectiveness of thyroid hormones for mitigating or alleviating psychiatric comorbidities in patients with acquired hypothyroidism

The role and use of THs in the treatment of psychiatric disorders have been well documented. While THs are prescribed to patients with hypothyroidism, they are also known to accelerate or enhance the efficacy of some psychiatric drugs, particularly in refractory patients without thyroid disorders (44, 45). Some of these therapeutic effects have been reported in patients who do not respond to antidepressant treatment, as well as in patients who require an accelerated effect of antidepressants. Multiple studies indicate that the administration of T_3_ hormone may benefit patients with depression (46–51). However, other reports suggest that the addition of L-T_4_ to antidepressant treatment may result in a more effective response to treatment (52–54).

Although depression is a disorder that often accompanies hypothyroidism, few reports have been conducted to determine whether the thyroid treatment reverses the depressive state in patients initially diagnosed with hypothyroidism. It has been reported that treatment of hypothyroidism with T_4_ alone does not improve mood (55). However, Gulseren et al. (2006) reported that hypothyroid patients with depression showed improvement after L-T_4_ administration, which reversed the hypothyroid state, without the need for psychiatric medication (28). Consistent with this, Rack and Makela (2000) reported a clinical case in which a patient with a long history of hypothyroidism treated with T_4_ maintained her depressed state, but when T_3_ was added to her treatment, her mood improved markedly (56). Several reports support the idea that the combination of T_4_+T_3_ prevents depression, although in some cases they report only a trend toward improvement (23, 57). In contrast, reports indicate that the combination of T_4_+T_3_ does not significantly improve depressive states in hypothyroid patients (58, 59).

Controversial evidence exists regarding the potential advantages of thyroid medication in reducing anxiety symptoms. The literature reports a benefit of adding L-T_4_ to anxiolytic treatment in hypothyroid patients with anxiety (28). Early initiation of L-T_4_ treatment is suggested to avoid anxiolytic administration (60). Monzani et al. (2023) reported that high doses of L-T_4_ were required to reverse anxiety in thyroidectomized patients (34). In contrast, Gunes et al. (2020) reported that anxiety may persist in hypothyroid patients even after achieving euthyroid status through thyroid medication (25).

Schizophrenia’s occurrence in hypothyroid patients, also known as myxedema psychosis, is rare. Thyroxine treatment’s efficacy has been reported in various clinical cases of this condition. A report indicates that T_4_ and T_3_ combination therapy (61) as well as T_4_ treatment alone improved symptoms in a woman with myxedema psychosis (62). Finally, there is no evidence indicating the effectiveness of L-T_4_ in treating bipolar disorder in patients who have been initially diagnosed with hypothyroidism.

**Figure 1:**
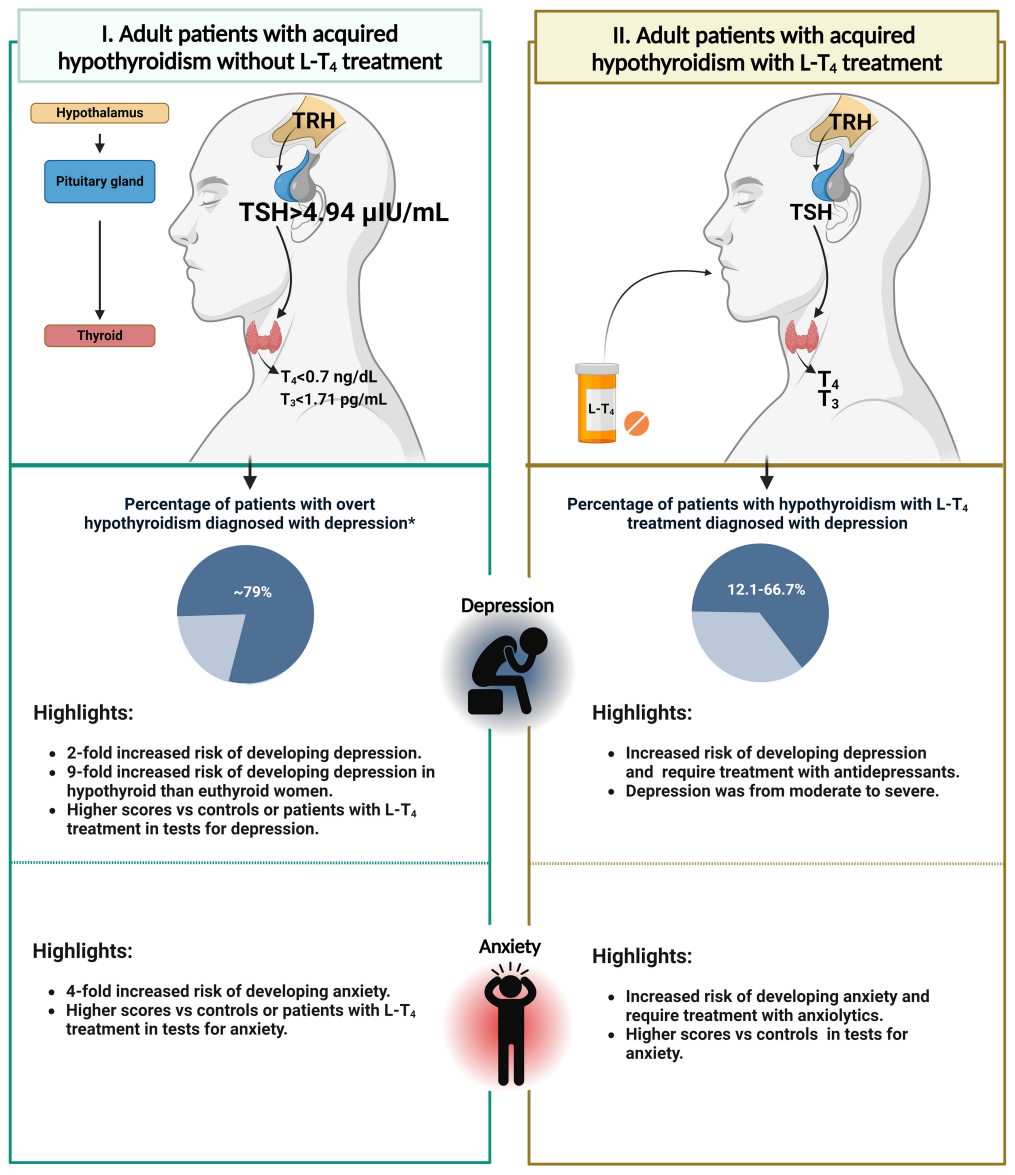
The association between acquired hypothyroidism and the subsequent development of depressive or anxiety disorders in adult patients. The figure on the left (I) depicts an adult patient with acquired hypothyroidism who has not received L-T_4_ treatment and their thyroid hormone levels (Usman et al., 2023). It is evident that there is a correlation between overt-acquired hypothyroidism and an increased risk of developing depression or anxiety. The figure on the right (II) shows an adult patient with acquired hypothyroidism who is undergoing L-T_4_ treatment. Furthermore, there is evidence indicating that depression or anxiety may be present despite the use of thyroid medication. The figure is based on clinical studies of the adult population (1, 21–26, 28–30, 33, 34). *Data obtained from the female population.

Determining the efficacy of THs in psychiatric disorders is challenging due to the varying results reported in studies. This variability can be attributed to differences in the severity of thyroid disease, pharmacological treatments, and the severity of their psychiatric conditions. Additionally, factors such as gender, age, disease onset, and duration may also affect results. It is important to note that the T_4_ hormone must be converted to T_3_ in various tissues, which can be a significant limitation in some patients, increasing the variability of reported results. However, because of the strength of the reports indicating the benefit of THs in improving the symptoms of psychiatric illness present in hypothyroidism, consideration should be given to adjusting the treatment of hypothyroidism to improve psychiatric symptoms, even though the reports are scarce and conflicting. **Figure 1**; **Table 1** summarize key information from this review.

## 8 Acquired hypothyroidism and depression: key points on mechanisms underlaying this relationship

Previous evidence has shown that acquired hypothyroidism is associated with the onset of depression. This finding has been confirmed by studies in rodents showing that adult-onset hypothyroidism induces depression-like behavior, as indicated by increased immobility time in the forced swimming test (FST) or tail suspension test (TST) (63–65). The literature from animal models and clinical studies indicates that acquired hypothyroidism may lead to depression through several mechanisms, including alterations in serotonergic neurotransmission or the hypothalamic-pituitary-adrenal axis (HPA axis), changes in adult hippocampal neurogenesis or brain region integrity, or by activation of systemic pro-inflammatory processes or neuroinflammation, or by the triggering of the kynurenine pathway. The following sections will provide a brief overview of the various mechanisms by which acquired hypothyroidism may lead to the development of depression.

### 8.1 Acquired hypothyroidism and serotoninergic neurotransmission

The monoamine hypothesis proposes that a reduced availability of monoamine neurotransmitters, such as serotonin (5-HT), dopamine and norepinephrine, results in decreased neurotransmission and impaired cognitive performance, which may lead to depression (66, 67). Preclinical and clinical evidence supports the notion that monoaminergic alterations could partially explain the presence of depression in patients with acquired hypothyroidism.

Although some studies have evaluated alterations in brain dopamine and norepinephrine levels in hypothyroid adult rodents, the results are controversial, and it is difficult to draw conclusions about them (63, 68–70). In contrast, the findings are consistent showing that serotoninergic neurotransmission is the most affected by hypothyroidism. Hassan et al. (2013) reported lower 5-HT levels in the blood plasma of hypothyroid rats in comparison to controls (68). Interestingly, serotonergic deficits are also observed in the brain. Yu et al. (2015) reported reduced 5-HT concentrations in the whole brain of hypothyroid rodents (71). More specific studies have shown that 5-HT is reduced in brain regions implicated in depression, such as the cerebral cortex (68), hippocampus (63, 69), prefrontal cortex (63, 69), and dorsal raphe nucleus (65), in adult rodents with hypothyroidism. Similarly, clinical evidence has demonstrated that patients with acquired hypothyroidism (without L-T_4_ treatment) exhibit lower platelet 5-HT concentrations (72); this deficiency is also observed in serum samples from hypothyroid patients receiving L-T_4_ treatment (73). Additionally, patients with acquired hypothyroidism have reduced central 5-HT activity, as evaluated by the response to dexfenfluramine (74, 75).

The expression of 5-HT_2A_ receptors in the prefrontal cortex (64, 76) and hippocampus (64) is lower in hypothyroid rats than in controls. Lee et al. (2017) reported that hypothyroidism provokes increased expression of 5-HT_1A_ receptors in the hippocampus (77), while Kulikov and Jeanningro (2001) did not observe changes in this receptor (76).

A recent study reported a significant inverse correlation between depression-like behavior (as measured by increased immobility time in the forced swim test or tail suspension test) and 5-HT levels or the expression of 5-HT_2A_ receptors in the prefrontal cortex and hippocampus of adult rodents with hypothyroidism (63, 64). At the clinical level, higher scores on the test for depression (test: HDRS) are correlated with reduced central 5-HT activity in patients with acquired hypothyroidism (75).

Thus, the evidence suggest that acquired hypothyroidism induces a deficit in serotoninergic neurotransmission by reducing the 5-HT concentration and the expression of postsynaptic 5HT_2A_ receptors. Consequently, these changes could lead to the development of depressive symptoms in patients. More studies should be carried out to elucidate the role of dopamine and norepinephrine in the relationship between hypothyroidism and depression.

### 8.2 Acquired hypothyroidism and kynurenine pathway

The kynurenine pathway (KP) plays a pivotal role in the pathophysiology of depression and has also been linked to hypothyroidism. KP metabolites play important roles in various tissues, either as a neuroprotective agent (kynurenic acid) or a neurotoxic agent (quinolinic acid). Additionally, KP modulates the immune system and provides energy in the form of nicotinamide adenine dinucleotide (NAD) for immune system responses. In the context of psychiatric disorders, numerous KP metabolites exhibit neuroactive properties, modulating neuroplasticity and exerting neurotoxic effects, at least in part, through their influence on NMDA receptor signaling and glutamatergic neurotransmission. In consequence, these metabolites have been demonstrated to have a significant association with psychiatric illness in the context of inflammation (78).

The KP is initiated by the metabolism of tryptophan (TRP). It is noteworthy that TRP is also the precursor of 5-HT and NAD, which is a cofactor involved in various metabolic pathways. In the TRP metabolism, the enzyme that represents the limiting step is indoleamine 2,3-dioxygenase (IDO). This enzyme determines whether the kynurenine or serotonin pathway or NAD synthesis occurs. IDO catalyzes the conversion of TRP into kynurenine (KYN), which is converted into two metabolites: 3-hydroxykynurenine (3-HKYN) or kynurenic acid (KYNA). Subsequently, 3-HKYN is transformed into 3-hydroxyanthranilic acid (3-HAA), and it gives rise to quinolinic acid (QUIN), which acts as an agonist of NMDA receptors and may induce excitotoxicity and neuronal death (79).

A study of 57 young women with autoimmune thyroiditis revealed an abnormal activation of KP in these patients in comparison to healthy controls. The levels of KYN and anthranilic acid (AA) were elevated, while KYNA was reduced in autoimmune thyroiditis, resulting in an imbalance between AA and KYNA levels. In contrast, the other metabolites, including 3-HKYN and 3-HAA, remained unchanged, whereas QUIN exhibited a slight increase in patients with autoimmune thyroiditis (80).

The evidence from research in humans and animal models indicates that KYNA deficiency can result in neuronal loss (81, 82), while increased KYNA in the brain can induce cognitive dysfunction due to reduced signaling through NMDA receptors, which are critical for learning and memory (83, 84). Thyroid hormones modulate the synthesis of the tryptophan metabolite, KYNA. Experimental hypothyroidism is associated with an independent increase in cerebral KYNA levels. Some data indicates the existence of a new mechanism related to thyroid hormone deficiency. This suggests that high KYNA levels may play a role in cognitive impairment associated with hypothyroidism (85).

In pathological conditions, nitric oxide (NO) can cause oxidative damage through the formation of the highly reactive metabolite peroxynitrite. NO is synthesized from L-arginine by nitric oxide synthase (NOS). There are different isoforms of NOS, and both the endothelial (eNOS) and neuronal (nNOS) isoforms are calcium-dependent, while the inducible isoform (iNOS) is calcium-independent (86). It has been postulated that an elevation in reactive oxygen species (ROS) levels, resulting from the deficiency of thyroid hormones, may give rise to an oxidative stress condition in various organs, including the brain, with a subsequent lipid peroxidation response (85).

In a model of hypothyroidism induced by the administration of methimazole in adult rats, an increase in oxidative stress was found in the hippocampus and amygdala. This was accompanied by increased levels of free radicals, lipid peroxidation and nNOS activity (87). This oxidative stress was accompanied by the triggering of the apoptotic pathways and neuronal damage in all regions of the hippocampus (88), an effect that is mediated by the overactivation of NMDA receptors (89). These findings indicate that adult-onset hypothyroidism results in oxidative stress that leads to neuronal death in the hippocampus, where the nitrergic system is involved. This provides a potential explanation for the behavioral abnormalities observed during hypothyroidism.

The activation of the KP, the overproduction of NO, and the subsequent oxidative stress in hypothyroid subjects indicate that QUIN may be a potential mediator between acquired hypothyroidism, immunological and neurotransmitter alterations, and depression. This hypothesis is based on the observation that pro-inflammatory cytokines induce a change in 5-HT synthesis due to alterations in tryptophan metabolism by the activation of the kynurenine pathway in glial cells, which may ultimately lead to 5-HT depletion and increased production of the neurotoxic metabolite QUIN (90). Collectively, these processes may result in the onset of depression.

Based on the above, the potential causes of depressive symptoms in patients with acquired hypothyroidism include the triggering of the KP, induced oxidative stress, overstimulation of NMDA receptors, and neuronal apoptosis. It is possible that these events may be provoked by an overproduction of QUIN, although this hypothesis requires further investigation. Furthermore, the depletion of 5-HT caused by the activation of the kynurenine pathway also plays a significant role in the onset of depressive symptoms.

### 8.3 Acquired hypothyroidism and hypothalamic pituitary adrenal axis

The HPA axis is a vital neuroendocrine circuit responsible for regulating a variety of physiological processes and responses to stress (91). This axis coordinates the secretion of glucocorticoid hormones, such as cortisol, under normal conditions and in reaction to stressors (92). The HPA axis also facilitates the physiological adaptations that occur during stressful situations. Its activity is regulated through negative feedback mechanisms, which allow for self-regulation (93).

Dysregulation of the HPA axis has been documented in patients with acquired hypothyroidism, who have elevated serum cortisol levels (94, 95). Sinha et al. (2023) reported that patients with acquired hypothyroidism had significantly higher serum cortisol levels compared to healthy controls. They also found a positive correlation between raised serum cortisol and raised TSH levels; and a negative correlation between high serum cortisol and lower T_3_ and T_4_ levels. The authors explain that elevated cortisol levels in patients with hypothyroidism may be due to reduced cortisol elimination and negative cortisol feedback in the HPA axis. However, it could also be a possible compensatory mechanism triggered by the HPA axis with increased cortisol secretion to mitigate the metabolic consequences of thyroid hormone deficiency (94).

On the other hand, it has been reported that dysregulation of the HPA axis can lead to various physiological and psychological alterations, which in turn lead to the development of mood disorders (96). This is important because it shows that hypothyroidism affects the HPA axis and can cause depression. The HPA axis is implicated in the pathogenesis of conditions such as depression and post-traumatic stress disorder. Patients with both disorders exhibit abnormalities in the HPA axis regulation, including altered cortisol levels and a failure to suppress cortisol release in the dexamethasone suppression test (97). Hernandez et al. (2008) reported hypercortisolism (measured in urine) in patients with depression compared to healthy volunteers. Only after 52 weeks of treatment with selective serotonin reuptake inhibitors (SSRIs) did the patients achieve a significant reduction in their cortisol levels; however, the authors considered this reduction to be only a partial recovery of HPA axis function (98). Although patients improved clinically in terms of depressive symptoms, elevated cortisol levels resulting from HPA axis dysregulation kept potential relapse latent. Furthermore, in animal models, dysregulation of the HPA axis has been demonstrated to result in an increased depression-like behavior (99). In normal conditions, cortisol serves to reduce inflammation. However, if an individual is chronically stressed or exhibits a dysregulation of the HPA axis, the subjects will present chronic hypercortisolism. This results in a diminished capacity of the HPA axis to regulate itself, which can lead to the release of pro-inflammatory cytokines by immune cells (100). Elevated pro-inflammatory cytokines have been demonstrated to play a significant role in the pathophysiology of depression (101).

Collectively, the evidence indicates that the development of depression in patients with acquired hypothyroidism appears to be significantly correlated with dysregulation of the HPA axis. This is consistent with the high prevalence of depression in hypothyroid patients and the reports of patients with depression with alterations in the HPA axis. Further research in patients with acquired hypothyroidism, with particular attention to HPA axis function and depressive symptoms, is essential to fully understand this complex relationship between thyroid function, the HPA axis, and depression. In conclusion, chronic hypercortisolism due to HPA axis dysregulation in hypothyroid patients appears to be key to the development of depression in these patients.

### 8.4 Acquired hypothyroidism and pro-inflammatory cytokines

Patients with hypothyroidism frequently present alterations in proinflammatory cytokines. Studies have demonstrated that individuals with hypothyroidism exhibit elevated levels of proinflammatory cytokines, including TNF-α, IL-6, and C-reactive protein (CRP), when compared to healthy individuals (102). It has been observed that levothyroxine treatment can reduce these cytokine levels, although it is not always possible to achieve normalization (102).

A study conducted in 2006 on patients with Hashimoto thyroiditis demonstrated a significant positive correlation between elevated serum IL-6 levels (a pro-inflammatory cytokine) and the required L-T_4_ dose. Conversely, a significant negative correlation was observed between elevated IL-6 levels and serum T_3_ and T_3_/T_4_ ratio (103). Another study in patients with autoimmune thyroiditis found that the serum levels of IL-2, IFN-γ, and TNF-α were elevated. This increase in these three pro-inflammatory cytokines may be explained by a higher number of activated T cells, resulting from the recognition of thyroid autoantibodies (104). Figueroa-Vega et al. (2010) demonstrated that patients with Hashimoto thyroiditis exhibit elevated circulatory levels of T-helper 17 (Th17) cells, a subpopulation of proinflammatory lymphocytes. Furthermore, elevated serum levels of IL-6 and IL-15 were observed in these patients. Both cytokines are involved in the differentiation of Th17 cells and possess pro-inflammatory properties (105).

The presented evidence highlights the pro-inflammatory state observed in hypothyroid patients. Indeed, Lai et al. (2024) propose a bidirectional relationship between elevated pro-inflammatory cytokines and the observed alterations in thyroid function (102).

Therefore, it is inevitable to associate depression in this context, a disorder characterized predominantly by a proinflammatory state. Liu et al. observed that patients with depression presented elevated serum levels of IL-1β, which correlated positively with the severity of depressive symptoms. In addition, TNF-α emerged as a promising biomarker for predicting the elevated risk of suicidal behavior (106). It is well established that systemic inflammation can affect the permeability and normal function of the blood-brain barrier (107), allowing peripheral cytokines to access the brain and producing neuroinflammation and alterations in neurotransmitter function (108). Furthermore, cytokines may influence monoamine synthesis through the degradation of tetrahydrobiopterin (BH4), a cofactor essential for 5-HT synthesis (108).

In 2017, Tayde et al. (2017) raised the question of whether proinflammatory cytokines are the link between hypothyroidism and depression (109). In this intriguing study, the researchers observed that individuals with primary autoimmune hypothyroidism exhibited elevated levels of IL-6, TNF-α, and CRP. The study included patients with antithyroid antibodies and TSH levels ≥10 μIU/mL. Following six months of treatment with levothyroxine, 42% of patients achieved remission of depression and an euthyroid state. Moreover, the administration of levothyroxine resulted in a significant reduction in the levels of the three pro-inflammatory markers, yet the reduction did not reach baseline levels (109). The findings of Tayde et al. (2017) provide compelling evidence that restoring thyroid function through L-T_4_ treatment in hypothyroid patients can alleviate depressive symptoms while reducing the proinflammatory state in these patients. This study demonstrates that proinflammatory cytokines are crucial in the pathophysiology and mechanism of the development of depression.

It is crucial to acknowledge that most of the studies discussed in this section were conducted in patients with autoimmune thyroiditis. It would be valuable to ascertain whether the proinflammatory state observed in this form of hypothyroidism is also present in other types of hypothyroidism acquired through non-autoimmune causes.

### 8.5 Acquired hypothyroidism and adult hippocampal neurogenesis

Adult neurogenesis is the generation of new neurons in the mature brain (110). In the hippocampus, adult neurogenesis occurs in the subgranular zone of the dentate gyrus (111, 112) and is a complex process that involves the proliferation of neural stem cells and progenitor cells (Type 2a, 2b and 3 cells) (113–115), the differentiation of neuroblasts into granular neurons through the stadium of immature granular neurons (IGNs), and the integration of new neurons into preexisting circuits in the dentate gyrus (116, 117). It has been postulated that reduced adult hippocampal neurogenesis could be implicated in the pathophysiology of depression (118, 119), principally by leading to overactivation of the hypothalamus-pituitary-adrenal axis (120, 121) and by affecting the contextual encoding of emotions (119, 122).

Evidence from animal models has shown that adult-onset hypothyroidism provokes impaired neurogenesis, exerting a deleterious effect, particularly on postmitotic cells. Specifically, hypothyroidism significantly reduces the population of quiescent Type 2b and 3 cells, postmitotic neuroblasts and IGNs (123–126). This reduced neurogenesis appears to be mediated by TRα1 aporeceptors (unlinked receptors), which could be predominant in hypothyroidism and be capable of repressing the expression of proneural and cell survival genes (127–129).

Interestingly, the neurogenic deficit in hypothyroid adult rats is accompanied by decreased expression of brain derived neurotrophic factor (BDNF) in the whole hippocampus (130) and, particularly, in the dentate gyrus (126). This phenomenon has also been reported in patients. Bilous et al. (2020, 2021) reported decreased expression of BDNF and neurogenesis-regulated genes in patients with primary hypothyroidism (131, 132). Alterations in the expression of BDNF are relevant because of three reasons: 1) the BDNF promoter is responsive to thyroid hormones (133), 2) this neurotrophic factor plays a key role in the postmitotic phase of the neurogenic process, promoting the survival of newborn neurons (134, 135), and 3) several studies have highlighted associations between low levels of BDNF and the development of behavioral symptoms of depression (136). Thus, BDNF could be the link between adult hypothyroidism, reduced neurogenesis and depression.

The relationship between adult neurogenesis and depression-like behavior in hypothyroidism condition was explored by Montero-Pedrazuela et al. (2006), who reported that reduced neurogenesis is accompanied by increased immobility time in the FST in hypothyroid rats (125). In contrast, increased hippocampal neurogenesis, provoked by simultaneous treatment with T_3_ and fluoxetine, is associated with antidepressant behavior in rats (test: novelty suppressed feeding test) (137).

This evidence suggests that acquired hypothyroidism can induce depression by reducing adult neurogenesis and BDNF expression in the hippocampus. Deeper studies should be carried out to determine the causal relationships and molecular pathways involved in the associations between hypothyroidism, neurogenesis and depression.

### 8.6 Acquired hypothyroidism and structural changes in brain

Because thyroid hormones play an important role in the development of the nervous system, it is important to study whether structural changes are present in patients with acquired hypothyroidism. Although the adult brain has completed its development, thyroid hormones maintain different functions on neuronal physiology, so hypothyroidism may cause changes in the metabolism and structure of the nervous system, causing alterations in the functionality of different brain regions. Currently, with the development of various brain imaging techniques, it has been possible to study possible changes in brain structure in patients with untreated acquired hypothyroidism (138).

One structure that has received particular attention is the hippocampus, as this structure is associated with learning and memory functions as well as mood states, functions that are clearly altered in hypothyroid patients. There are reports in animal models of damage to the pyramidal neurons of the hippocampus as a result of decreased thyroid hormone levels (139, 140).

Using magnetic resonance imaging (MRI) in patients with untreated acquired hypothyroidism, a reduction in hippocampal volume has been observed (141), as well as a reduction specifically in the granular and molecular layers of the hippocampus (142). It has been proposed that the volume reduction correlates with a decrease in peripheral BDNF levels (143). In addition, using positron emission tomography (PET), it has been shown that hippocampal glucose utilization is reduced in hypothyroid patients (144).

Other structures associated with psychiatric disorders, such as the prefrontal cortex or the amygdala, have also been studied for changes due to the effects of acquired hypothyroidism. Although there are fewer studies on this topic, there is evidence of structural and metabolic changes, although the results are more variable.

Using voxel-based morphometry, a reduction in gray matter in the cerebellum as well as white matter in the cerebellum, frontal gyrus, temporal gyrus, and occipital gyrus has been reported (145), while using MRI, the group of Leyhe et al. (2014) observed decreased gray matter in the inferior frontal gyrus (146).

Regarding metabolic changes, decreased glucose utilization in the amygdala and anterior cingulate cortex has been reported (144), as well as decreased blood flow in the prefrontal, temporal and occipital lobes (147).

All these structural changes observed in hypothyroid patients could affect the proper functioning of the central nervous system and contribute to the development of psychiatric disorders.

## 9 Conclusion

Evidence supports that acquired hypothyroidism increases the risk of depression and anxiety in adult patients. Current data indicates that patients with acquired hypothyroidism without L-T_4_ treatment face a 2-fold increased risk of depression and a 4-fold elevated risk of anxiety, with a notably higher risk for depression in women (about 9-fold more risk). The prevalence of depression in these patients is 79%, and they show elevated scores in depression or anxiety tests when compared to controls or patients receiving L-T_4_ treatment. On the other hand, the literature shows that both depression and anxiety can be also present in patients with acquired hypothyroidism with L-T_4_ treatment. These patients are at a higher risk of developing depression or anxiety and therefore require treatment with antidepressants or anxiolytics. Depression affects between 12.1% and 66.7% of these patients and is often classified as moderate to severe. Regarding anxiety, these patients exhibit higher scores on tests for anxiety versus controls.

The significant prevalence of depression in patients with acquired hypothyroidism, coupled with the high susceptibility to developing depression and anxiety, highlights the need for enhanced monitoring of psychiatric symptoms. Consistent monitoring and timely recognition of psychiatric symptoms, especially in patients with TSH serum levels >2.5 µIU/mL, will prevent disease aggravation, facilitating more effective therapeutic management. On the other hand, there is no strong evidence suggesting that acquired hypothyroidism could be a risk factor for schizophrenia or bipolar disorder; however, more scientific research is necessary to address this matter. Finally, key points on the underlying mechanisms of the relationship between hypothyroidism and depression are presented. These evidence the different ways in which acquired hypothyroidism can lead to the onset of depression. These include disturbances in serotonergic neurotransmission or the HPA axis, alterations in adult hippocampal neurogenesis or brain region integrity, activation of systemic pro-inflammatory processes or neuroinflammation, or by the triggering of the kynurenine pathway.

## Author contributions

NO-B: Writing – original draft. AV-P: Writing – original draft. DS: Writing – original draft. YG-M: Writing – original draft. JP-R: Writing – original draft. GP-S: Writing – review & editing, Supervision, Project administration, Conceptualization, Writing – original draft. KS-H: Writing – review & editing, Supervision, Project administration, Funding acquisition, Conceptualization, Writing – original draft.
