# Supplementary material for: Is the acquired hypothyroidism a risk factor for developing psychiatric disorders?
Source: Front Psychiatry. 2024 Jul 19;15:1429255. doi: 10.3389/fpsyt.2024.1429255 (PMC11294236; doi:10.3389/fpsyt.2024.1429255)
Supplement: Supplementary file 1 [file DataSheet_1.zip › Methods.docx]

Supplementary material

*II. Methods*

Search criteria. PubMed, Scientific Electronic Library Online (SciELO), and Google Scholar databases were utilized for the literature review. The review included scientific articles in both English and Spanish without any restrictions regarding publication dates. The keywords include acquired hypothyroidism, overt hypothyroidism, clinical hypothyroidism, autoimmune thyroiditis, Hashimoto’s thyroiditis, chronic autoimmune thyroiditis, depression, major depressive disorder, affective disorder, anxiety, anxiety disorder, schizophrenia, levothyroxine, L-T_4_, thyroid medication, adult-onset hypothyroidism, serotonin, 5-HT, serotonin receptors, dopamine, norepinephrine, kynurenine pathway*,* kynurenic acid, quinolinic acid, oxidative stress, neuronal apoptosis, HPA axis, cortisol, pro-inflammatory cytokines, inflammation, adult hippocampal neurogenesis, BDNF, Type 1 cells, Type 2b cells, Type 3 cells, immature granular neurons, hippocampus, prefrontal cortex, amygdala, MRI and PET. Literature selection criteria. Scientific articles were selected from two groups of patients: Study population 1: adult population with a mean age ≥18 diagnosed with acquired hypothyroidism and without thyroid medication. It should be noted that acquired hypothyroidism was diagnosed based on high serum levels of TSH (usually >4µIU/mL) and low serum levels of T_4_ (usually <0.7 ng/dL) in comparison with the reference values for each population. Study population 2: adult population with a mean age ≥18 diagnosed with acquired hypothyroidism and with thyroid medication (with evidence of medical prescription of thyroid treatment or with thyroid profile showing an euthyroid state due to L-T_4_ treatment). For Section VIII, studies in rodents with adult-onset hypothyroidism were also included. Exclusion criteria. We excluded: 1) clinical reports of psychiatric disorders in patients with other types of hypothyroidism, such as subclinical hypothyroidism, endemic cretinism, congenital hypothyroidism, and maternal hypothyroxinemia; 2) articles not specifying the type of hypothyroidism, 3) clinical studies of patients selected exclusively by their TSH or anti-thyroid antibodies serum levels, and 4) articles of patients diagnosed with psychiatric disorders before hypothyroidism. For Section VIII, studies in rodents with developmental hypothyroidism or maternal hypothyroxinemia were excluded. Limitations. References in section VI: Sharif et al. 2018; Benros et al., 2011 and Thomsen et al., 2005 (41-43), do not fully meet the selection criteria. Their inclusion was due to the lack of literature in this field, and these papers show a first approach to this topic.
